# Skin-derived stem cells as a source of primordial germ cell- and oocyte-like cells

**DOI:** 10.1038/cddis.2016.366

**Published:** 2016-11-10

**Authors:** Wei Ge, Shun-Feng Cheng, Paul W Dyce, Massimo De Felici, Wei Shen

**Affiliations:** 1 Institute of Reproductive Sciences, Key Laboratory of Animal Reproduction and Germplasm Enhancement in Universities of Shandong, College of Animal Science and Technology, Qingdao Agricultural University, Qingdao 266109, China; 2 Department of Animal Sciences, Auburn University, Auburn, AL 36849, USA; 3 Department of Biomedicine and Prevention, University of Rome ‘Tor Vergata', Rome 00133, Italy

## Abstract

The skin is a unique organ that contains a variety of stem cells for the maintenance of skin homeostasis and the repair of skin tissues following injury and disease. Skin-derived stem cells (SDSCs) constitute a heterogeneous population of stem cells generated *in vitro* from dermis, which can be cultured as spherical aggregates of cells in suspension culture. Under certain *in vitro* or *in vivo* conditions, SDSCs show multipotency and can generate a variety of neural, mesodermal, and endodermal cell types such as neurons, glia, fibroblasts, adipocytes, muscle cells, chondroblasts, osteoblats, and islet β-cell-like cells. SDSCs are likely derived from multipotent stem cells located in the hair follicles that are, in turn, derived from embryonic migratory neural crest or mesoderm cells. During the past decade, a wave of reports have shown that germ cells can be generated from various types of stem cells. It has been shown that SDSCs are able to produce primordial germ cell-like cells *in vitro*, and even oocyte-like cells (OLCs). Whether these germ cell-like cells (GCLCs) can give rise to viable progeny remains, however, unknown. In this review, we will discuss the origin and characteristics of SDSCs from which the GCLC are derived, the possible mechanisms of this differentiation process, and finally the prospective biomedical applications of the SDSC-derived GCLCs.

## Facts

- A variety of stem cells for the maintenance of skin homeostasis and the repair of skin tissues reside in the skin stem cell niches.
- Many studies indicate that SDSCs showed multiple differentiation repertoire *in vitro* and may even give rise to germ cells.
- Murine SDSC-induced OLCs showed the robust ability to restore estradiol production and estrus cycling when transplanted under kidney capsule of ovariectomized mice resembling their normal *in vivo* counterparts.

## Open Questions

- Where are these SDSCs originated during early embryogenesis?
- Why SDSCs with limited differentiation potential showed surprising differentiation repertoire *in vitro*?
- What do we know about germ cells from skin?
- Whether artificial germ cells induced from SDSCs can be used as candidates for human infertility and premature ovarian failure treatment?

**Figure 1:**
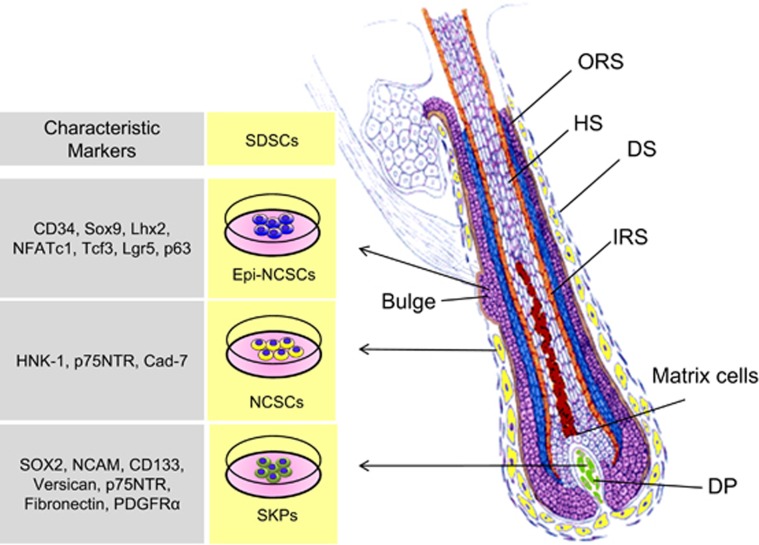
Diagram of the hair follicle structure depicting the origin of the three main types of SDSCs isolated *in vitro*

Skin is one of the few organs in mammals that continuously self-renews most of its tissue components (epidermis and dermis) and structures (hair follicles, sebaceous glands, and sweat glands), even into adulthood. This extraordinary property is because of the presence of various types of stem cells located in the epidermis, dermis, and the hair follicles.^1^ Previous studies indicated that a steady flow of differentiating keratinocytes in the epidermis supplied by relatively rare epidermal stem cells, located in the basal layer and in the deep rete ridges of the epidermis, are responsible for epidermis homeostasis;^2, 3, 4^ however, recent studies indicated that basal epidermal cell constitute an equipotent pool of progenitors in the murine ear and paw epidermis.^5^ Moreover, mesenchymal stem cells, characterized by typical mesenchymal markers and with multipotent differentiation capacity, are localized in the dermis, while melanocytes stem cells, able to produce new melanocytes, are present in the hair follicles.^6, 7^ In hair follicles a population of stem cells reside in a discrete microenvironment called the bulge (Figure 1). The bulge region is located at the base of the hair follicle, established during morphogenesis, and does not degenerate during the hair growth cycle.^8, 9^ During the hair cycle, the bulge stem cells are stimulated to exit their niche, proliferate, and differentiate to form the various cell types of a mature hair follicle.^10, 11^ In addition to the bulge stem cells, the hair follicle contains dermal stem cells that orchestrate the hair regeneration and repair of skin tissues and structures following injury and disease.^12^ Dermal stem cells are localized in the dermal sheath (DS), probably in the bulge itself but mainly in the dermal papilla (DP) (Figure 1).^13, 14^

Hair follicles are composed of an outer root sheath (ORS) of epidermal cells that are contiguous with the epidermis, an inner root sheath (IRS) of connective tissue, and the hair shaft (HS) (Figure 1). The hair follicle cycle involves three stages: telogen (resting), catagen (regression), and anagen (growth).^15, 16^ During catagen, the lower two-thirds of the follicle gradually disappears and the DP reaches the level of the bulge. When the next round of anagen begins, daughter cells derived from the bulge stem cells move onto the DP and become new matrix cells reinitiating an HS. The DP seems to be a key niche component and a source of signals that stimulate the activity of matrix cells.^17, 18^ Hair follicles do not develop, persist or function without DP. Along with the DP, another dermal component of the hair follicle is the DS. The DS lines the epithelium of the hair follicle from the bulge level downward and is contiguous with the base of the DP through a stalk. DP and DS are separated from the epithelial portion of the hair follicle by a basement membrane. The DS consists of three layers of collagen fibers running in different directions containing fibroblasts mostly residing in the thickened middle collagen layer. Cells within DP and DS possess stem cell features and are likely derived from the embryonic neural crest (NC) and/or mesoderm cells.

Many laboratories have independently described the *in vitro* isolation of multipotent cells from human, pig, and rodent skin with stem cell properties termed skin-derived stem cells (SDSCs).^19, 20, 21, 22^ These cells can survive and grow, *in vitro*, as spheres in suspension culture and appear to be derived from different structures within the hair follicle.^23, 24, 25^ On the basis of their expression of genetic markers among other characteristics, SDSCs obtained *in vitro* may be subdivided into at least three cell types: NC stem cells (NCSCs) derived from cells located in the DS, epidermal NCSCs derived from cells of the bulge, and skin precursor cells (SKPs) derived from cells of the DP (Figure 1). The cell populations of the facial hair follicles are capable of forming SDSCs that originate from embryonic NC cells, whereas those of the trunk hair follicles are presumably of both NC and mesodermal origin (http://www.stembook.org/node/696.html). In any case, the potential of all types of SDCS to generate neurons, glia, myofibroblasts, chondrocytes, adipocytes, and melanocytes *in vitro* indicates a considerable genome plasticity, resembling that of the embryonic NC cells. Over the past decade, studies have shown that SDSCs may have a broader developmental potency than previously expected, among which is their potential to generate germ cell-like cells (GCLCs). These observations are of particular interest as these SDSC-derived GCLCs may be potential candidates for treating human infertility and premature ovarian failure (POF). The present review discusses the developmental potential of SDSCs to differentiate into GCLCs and summarizes recent research advances using SDSCs as a model to investigate the differentiation potential of GCLCs from adult stem cells (ASCs). Finally, a discussion of current research progress and potential biomedical applications of the SDSC-derived GCLCs are reported.

### SDSCs derive from stem cell populations originated from multipotent embryonic NC or mesodermal cells

In the mouse embryo, epidermal differentiation can be traced back to E8.0 (embryonic day), when the transcription factor p63 is expressed in the single layer of ectoderm cells surrounding the embryo and determines the epidermal fate.^26^ The precursors of the hair follicles are present in a local thickened region of the embryonic epidermis, known as the placode, which is detectable at E14.5. Reciprocal signaling between the placode and the condensate leads to proliferation of the overlying epithelium and downward extension of the developing follicle into the dermis.^27^ Following the downward growth, the epithelial cells envelope the dermal condensate forming the DP.^14^ The DP in hair follicles located at different sites of the body have different embryonic origins,^28, 29, 30, 31^ in the head and face region they are derived from NC cells, whereas in the dorsal and ventral trunk skin they originate from the dermomyotome of somite and lateral plate origin, respectively, with probable contribution of NC-originating cells. Between E14.5 and E16.5, all developing DP contain cells expressing the transcription factor sex determining region Y-box 2 (SOX2); however, SOX2 remains undetectable in the DP of 'zigzag' hairs (the thinnest mouse hair type), which develop from E18.5 onwards.^32^ In the adult SOX2^+^ cells remain mainly in the DP and constitute a reservoir of dermal stem cells. These cells appear to maintain the multipotency of their NC cell progenitors, and are considered a transient and multipotent embryonic stem cell (ESC) population also termed NCSCs.

**Figure 2:**
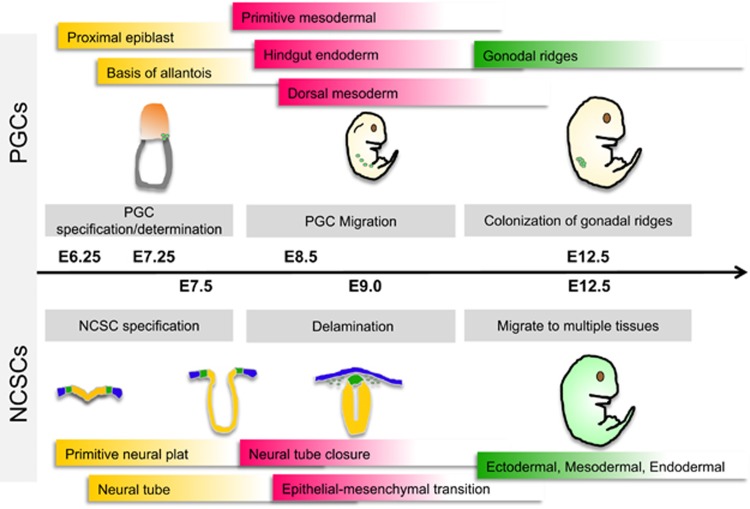
Main developmental stages of PGC and NCSC in the mouse embryo

NCSCs derive from the neural tube (Figure 2) and are induced to migrate and give rise to various cell lineages: melanocytes, craniofacial cartilage, bone, smooth muscle, peripheral and enteric neurons, and glia cells. *In vitro*-produced SDSCs can be differentiated into cell types that are highly reminiscent of NCSC-derived populations.^33^ In particular, Fernandes *et al.*^28^ demonstrated that among the different SDSC populations, SKPs possessed differentiation potential similar to NCSCs and that targeted SKPs showed migratory behavior resembling that of NCSCs when transplanted into the chick NC cell migratory stream. Moreover, *in vitro* studies have demonstrated that SKPs derived from SOX2^+^ cells located in the DP of the skin trunk can be differentiated into a variety of cell types including lineages that are never seen in normal skin *in vivo*, such as insulin-producing cells and germ cells.^24, 34^ Biernaskie *et al.*^14^ demonstrated that SKPs and SOX2^+^ hair follicle DP cells are similar with regard to their transcriptome and functional properties. Both SKPs and endogenous SOX2^+^ cells induced hair morphogenesis and homed to a hair follicle DP niche upon transplantation.^14^ In addition, rodent SKPs express several transcription factors (i.e. *slug*, *snail*, *twist*, *pax3*, and *sox9*) that are involved in the specification and migration of NC cells.^28, 35^ However, p75(NTR), which is widely used in the identification and isolation of NCSCs, was either not expressed or undetectable in rodent dorsal and facial SKPs,^28^ or in human neonatal foreskin SKPs.^36^ In contrast, multipotent SKP cells from human and mouse trunk skin coexpressed p75NTR and SOX10.^30^ In pigs, SKP cells were reported to express both pluripotency-related genes and NC cell markers, further demonstrating the NC origin of SDSCs.^37^

All these results demonstrate that the various types of SDSCs may have a common origin deriving from the embryonic NC cells colonizing the dermal condensates developing below the epidermis during midembryogenesis. It appears that SDSCs, and in particular SOX2^+^ SKPs, represent residual NCSCs in adult skin, whose developmental potential is restricted *in vivo* by the niche they occupy, but is revealed when cultured *in vitro*.

### The surprising differentiation repertoire of SDSCs

**Figure 3:**
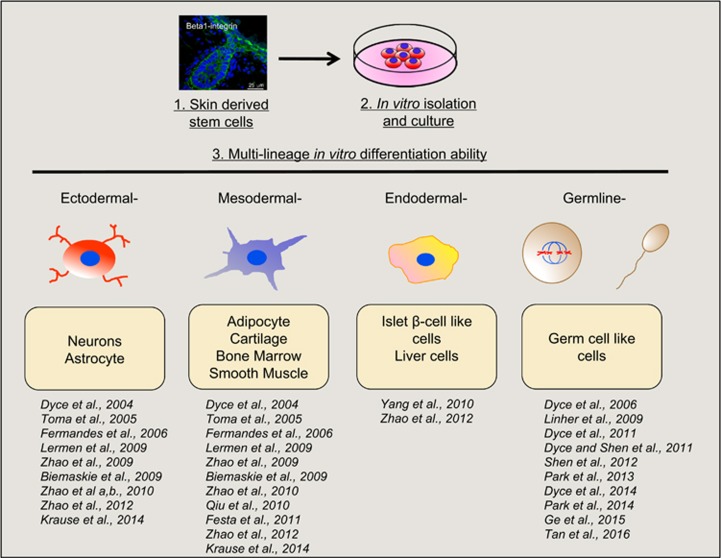
Multipotent differentiation potential of SDSCs

Contrary to the traditional view that ASCs are restricted to differentiating only into cell types belonging to their tissue of origin,^38^ SDSCs show a surprisingly wide differentiation repertoire (Figure 3). For example, dermal stem cells were capable of repopulating the hematopoietic system after transplantation into lethally irradiated recipient mice.^39^ Mouse SDSCs appear able to give rise to muscle progenitors and differentiated skeletal muscle cells when transplanted into injured muscles.^40^ Subcutaneous injection of SKPs into the dorsal skin of adult NOD/SCID mice resulted in cells that integrate into the interfollicular dermis and express dermal fibroblast markers.^14^ When YFP-labeled mouse SKP spheres were transplanted into the chick NC migratory stream *in ovo* at Hamburger-and-Hamilton stage 18, the sphere-derived cells migrated into the sympathetic ganglia, spinal nerve, dorsal root ganglion and even the dermal layer of the skin, whereas very few cells went into neural tube. Furthermore, Zhao *et al.*^41^ found that porcine SKPs injected into a morula were incorporated in the embryos and contribute to various somatic tissues of the three germ layers in postnatal chimera, and especially have an endodermal potency. Interestingly, GFP-positive cells were also observed in the gonadal ridges, although the identity of the positive cells was not determined. Finally, SKPs isolated from fetal porcine have been demonstrated to produce live offspring following nuclear transfer. The porcine SKPs were capable of long-term *in vitro* proliferation allowing for genetic modification before nuclear transfer into enucleated oocytes. The resulting cloned piglets show the ultimate potential of the SKPs to contribute to all cell types.^42^ These last findings are particularly intriguing as as we will discuss in detail in the next section, SDSCs isolated from fetal porcine and newborn mouse back skin possess germline potential *in vitro*.

Because SDSCs can be easily isolated from skin tissues, they are considerably more accessible than ESCs and less 'artificial' than induced pluripotent stem cells (iPSCs). Moreover, the use of SDSCs is not restricted by ethical issues and not subjected to immune rejection following autologous transplantation. Furthermore, SDSCs are highly proliferative (able to double their number within 3–4 days of culture) while maintaining their differentiation potential after long-term *in vitro* culture.^21^ Finally, they do not form tumors when transplanted in recipient hosts. As we will discuss in more detail below, these features together with the differentiation potential reported above make SDSCs an ideal stem cell population for use in stem cell-based therapies.

### Germ cell potential of SDSCs

In 2006, Dyce *et al.*^24^ demonstrated that oocyte-like cells (OLCs) could be obtained from SDSCs isolated from the fetal skin of both male and female pigs cultured in the presence of FBS and porcine follicular fluid (PFF).^24^ The authors, however, concluded that the *in vitro* culture system was inadequate to support the complete development of mature and competent oocytes. In others papers, the same group reported that pig SDSCs were able to produce cells similar to the primordial germ cells (PGCs), the precursors of oocytes, which were termed PGC-like cells (PGCLCs).^43^ The efficiency of PGCLC formation from SDSCs was quite low (~1.4%), like the subsequent derivation of OLCs (~1 out 1000 PGCLCs).^44^ Interestingly, the transfection of SDSCs with a deleted in azoospermia-like (DAZL) expression vector at the initiation of induced differentiation significantly enhanced the formation of PGCLCs (~4 times), and stimulated the expression of meiotic germ cell genes.^45^ Moreover, the heparin-binding growth factor midkine was found to promote the proliferation of SDSC-derived PGCLCs and that of endogenous PGCs as well.^46^ During differentiation, follicle-like structures were formed from the fetal SDSCs surrounding the OLCs.^24, 47^ Similar results were obtained from neonatal mouse skin, although the frequency of PGCLC formation was higher, ~7%.^25, 46, 48, 49^ Finally, mouse GFP^+^ OLCs aggregated with newborn ovarian cells, transplanted under the kidney capsule of ovariectomized recipient hosts, formed preantral and antral follicles.^15^

Other studies in the mouse, using modified protocols aimed to improve the efficiency of PGCLC production showed that embryoid body-like (EBL) formation from dissociated SDSC spheres was a necessary passage to generate GCLCs and that BMP4, activin A, and retinoic acid (RA) markedly increased the efficiency of the germ cell induction and stimulated PGCLC proliferation onto MEF monolayers.^43, 50^ Promising results have been recently reported in human, in which Ge *et al.*^51^ showed that SDSCs obtained from 4-month fetal skin, following procedures similar to that used for pig including the presence of PFF, could be differentiated into PGCLCs expressing the germ cell markers DAZL and the mouse Vasa homolog. Moreover, when cultured in media containing growth factors used to generate male germ cells from other types of stem cells (i.e. LIF, GDNF, RA), a few cells (~1%) appeared to form haploid cells by FACS analysis and showed punctate and elongated nuclear SCP3 staining suggesting meiosis occurrence.^51^

All these unexpected results generate several questions. In particular, the mechanisms underlying the generation of PGCLCs and OLCs and of the companion follicle-like cells. And certainly whether these cells are functional and if the OLCs generated can give rise to normal viable offspring.

Regarding the first question, a likely hypothesis is that a small sub-population of multipotent cells present in the skin described above when placed outside their niche in an appropriate environment first give rise to PGCLCs capable of then differentiating into OLCs. As we reported above, indeed SDSCs are likely derived from multipotent stem cells located in the hair follicles, which, in turn, derive from embryonic migratory multipotent NC or mesoderm cells. In this case, the culture conditions used to generate PGCLCs and OLCs from SDSCs should first recapitulate the process of PGC formation from the epiblast, and subsequently recreate the microenvironment for PGC differentiation into oocytes. Indeed, this is the same sequence of events described to occur spontaneously in the formation of PGCLCs and OLCs from mouse ESCs *in vitro* in the seminal work by Hübner and co-workers.^52^

From studies in the mouse, we know that during embryonic development the foundation of the germline is established by the specification of PGCs from the postimplantation epiblast by BMP and WNT3a signaling (Figure 3).^53, 54, 55^ PGCs initiate a unique cellular program driven by the cooperation of the transcription factors BLIMP1, PRDM14, and AP2*γ.*^56, 57^ Under appropriate culture conditions, cells from the mouse epiblast can give rise to self-renewing and pluripotent epiblast stem cells (EpiSCs) *in vitro.*^58^ These cells express BMP4 and continuously specify PGCLCs under self-renewing culture conditions, but at a low frequency (~2%).^59^ It appears that EpiSCs reflect a later developmental stage compared with that of when PGCs are initially specified *in vivo*, which could explain the low efficiency of PGC derivation *in vitro*. On the other hand, ESCs, which are derived from the preimplantation epiblast, can be differentiated *in vitro* into epiblast-like cells (EpiLCs) with activin A and bFGF, the same cytokines used to culture EpiSCs under self-renewing conditions.^60, 61, 62, 63^ EpiLCs in turn respond to BMP4 and give rise to functional PGCLCs at a high frequency (~35–40%).^64, 65^ Thus, it appears that stem cells must transit to a primed epiblast-like state initially, before they gain the competence to efficiently give rise to PGCs.^58, 66^

**Figure 4:**
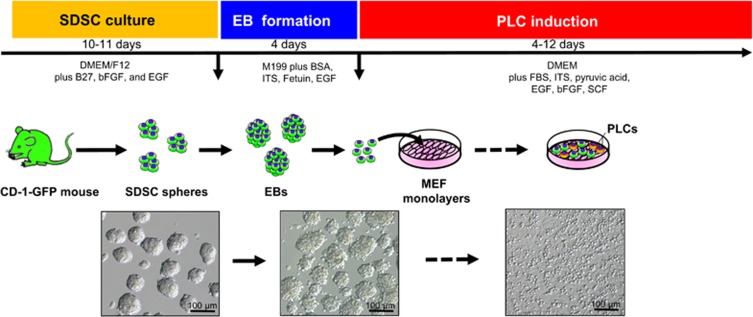
A schematic diagram of the main steps to produce mouse PGCLCs from SDSCs *in vitro*

The original procedures performed to generate PGCLCs and OLCs from pig and mouse SDSCs consist of three or two culture steps, respectively. The first step is the formation of SDSC spheres from dissociated skin cells in DMEM/F12 supplemented with B27, bFGF, and EGF. The dissociated sphere cells are then cultured for 30–50 days in the presence of DMEM supplemented with 5% FBS and 5% PFF, and finally the non-adherent aggregates with large cells (>50 *μ*m) are transferred for 5–14 days in M199 supplemented with BSA, ITS, pyruvic acid, fetuin, EGF, FSH, and LH. In the mouse, the dissociated sphere of SDSCs are simply cultured in this medium for 12 days to generate OLCs. Under such culture conditions, in both species, the efficiency of OLC formation was very low, 6–70 (maximum diameter about 100 *μ*m) out 50 000 plated SDSCs in pig and 10–50 (maximum diameter about 45 *μ*m) out of 600 000 SDSCs in mouse. We must postulate that compounds present in the differentiation medium induce SDSCs into EpiLCs and then into PGCLCs and these then into OLCs. Although none of the main components such as EGF, LH, FSH and BSA, insulin, transferrin, and fetuin were previously reported to have a critical role in germ cell formation, the analyses of global gene expression profiles and unpublished results (De Felici, personal communication) revealed that insulin or IGF-1 and the receptor for transferrin are both highly expressed in migrating PGCs,^67^ suggesting that they may have a role in early germ cell development. In addition, receptors for EGF, transferrin, and insulin were also found to be highly expressed in fetal mouse ovaries,^68^ implying a potential role of the signaling pathway in oogenesis. It is also possible that a small sub-population of the SDSCs spontaneously step onto the germ cell path after they are removed from their niche.^69^ The proliferation of these putative germ cells might be then stimulated by these factors, either individually or in combinations. In any case, as the frequency of PGCLC formation was low and the germline specification from SDSCs appears a quite inefficient process. This is probably due, at least in part, to the not optimal culture conditions used in the first studies to induce PGCLC specification/determination and to sustain their proliferation. Indeed, we found that in the mouse the frequency of PGCLC formation was markedly increased (from 7–8% to about 50%) when SDSCs were first induced to form EBL and then the dispersed EBL cells cultured onto MEF feeder layers in medium containing FBS, EGF, bFGF, and SCF. Moreover, the presence of BMP4, Activin A, or RA during the formation of embryoid bodies (EBs) and/or the culture onto the monolayer further increased the frequency of the percentage of PGCLCs up to 90% (Figure 4).^43^

An even more critical problem is that the current *in vitro* differentiation system does not support efficient PGCLC differentiation into OLCs or full OLC maturation. These problems are common to all procedures aimed at producing *in vitro* mature oocytes from stem cell-derived germ cells and indeed also from endogenous PGCs. In fact, *in vitro* culture conditions able to allow a natural germ cell to progress through meiosis forming a primary oocyte and to complete maturation remains to be devised. As it is known that PGCs and fetal primary oocytes undergo a conspicuous wave of apoptosis for various reasons, including the availability of certain growth factors and defects of meiosis,^70, 71^ it is possible that this process is exacerbated *in vitro*. Currently, meiosis entry and progression throughout the first meiotic division proves to be a difficult process to be correctly reproduced *in vitro*, and abnormal meiosis is frequently observed in stem cell-derived germ cells.^72, 73, 74, 75^ Only one paper reported complete meiosis *in vitro* from ESC-derived PGCLCs, and these PGCLCs showed similar erasure of genetic imprinting, chromosomal synapsis, and recombination resembling their *in vivo* counterparts; of great significance is that intracytoplasmic injection of these PGCLCs into oocytes resulted in viable and fertile offspring, demonstrating the complete gametogenesis *in vitro.*^76^ However, this observation raised great controversy among scientists working on germ cell biology and even Saitou *et al.* indicates that we have to be very cautious about the implications of this paper (http://www.nature.com/news/researchers-claim-to-have-made-artificial-mouse-sperm-in-a-dish-1.19453). In this regard, it should be mentioned that Dokshin *et al.*^77^ recently demonstrated that in the mouse oocyte differentiation is genetically dissociable from meiosis. In fact, these authors reported that a small number of oocytes from *Stra8* knockout mice survived and developed without any evidence of meiosis up to fully grown oocytes inside antral follicles. These oocytes were fertilized using *in vitro* fertilization and developed to the two-cell stage, but failed to develop further. These observations might explain the development of OLCs from SDSCs in culture reported by Dyce and by others from various types of stem cells but not their ability to function.^24^,^25^,^78^ Indeed, OLCs were often described to show some morphological features of oocytes such as spherical shape, relevant increasing volume, zona pellucida membrane formation and the expression of oocyte specific genes including *Figla*, *Nobox*, and *Bmp15*, but no evidence of correct meiosis.

In 2014, Handel *et al.*^73^ considered the production of chromosomally normal viable offspring as a 'golden standard' for *in vitro*-derived germ cells. As a matter of fact, the only method to achieve mature oocytes competent of being fertilized and giving rise to viable apparently normal pups from PGCLCs or endogenous PGCs was to reaggregate early OLCs or oocyte, respectively, with ovarian cells and to transplant the aggregates under the kidney capsule or ovarian bursa of recipients hosts.^56^ On the other hand, in the already mentioned most successful work that produced artificial gametes from mouse ESCs and iPSCs by Hayashi *et al.*,^64, 65^ only ~7 out of 1000 endogenous mouse PGCs and PGCLCs obtained from EpiLCs subjected to a similar transplantation procedure showed the capability to form mature oocytes. Nonetheless, it was less efficient to obtain pups from PGCLCs (~4.0%) than from 12.5 dpc PGCs or wild-type 3-week-old oocytes (~12.7% and 17.3%, respectively). Only recently, Zhou *et al.*^76^ reported that germ cell meiosis can begin and apparently be correctly completed *in vitro* in germ cells artificially obtained from stem cells. Indeed, these authors reported that haploid spermatids were obtained from male mouse EpiLCs-derived PGCLCs in 2 weeks and that such spermatid-like cells were capable of producing viable and fertile offspring after intracytoplasmic sperm injection.

### Possible biomedical applications of SDSC-derived germ cell-like cells

The first results describing the derivation of germ cell-like *in vitro* from stem cells and even live pups from such cells generated great excitement in both scientists and patients suffering with infertility.^79, 80, 81^ The ultimate aim to derive germ cells from stem cells is the production of viable normal offspring. This should certainly represent a turning point in reproductive medicine for a variety of infertility treatments.^82^ It is, however, important to point out that the science of artificial gamete technology is still in its infancy. Many of the scientific methods that can be used to create artificial gametes have never been experimentally accomplished in humans and still need further clinical investigation.^83^ Although much of this research has been carried out in mice, it has relevance for clinical application to humans. That said, at present the discovery of artificial gamete formation from stem cells is of most value for basic scientific research, whereas clinical applications remain only a hypothetical possibility.

In general, in order for a method to have applicative perspectives in regenerative medicine, it must be easily available, not require complex manipulation, be safe and efficient, and be considered ethically acceptable. The production of PGCLCs and OLCs from human SDSCs meet the first two characteristics, more study is required to determine if they are safe and efficient, although using SDSCs likely meets this criteria better than ESCs or iPSCs. The fourth criteria, to be ethically acceptable is less reachable, particularly concerning OLCs. Currently, the overall number of such cells obtained *in vitro* is quite variable and whether OLCs can be made meiotically competent and functional remains to be answered. The ultimate proof of course lays with the birth of viable, normal offspring generated from one such gamete. In this regard, the possibility to generate functional PGCLCs from male SDSCs has been investigated only in one paper^18^ and certainly requires further investigation.

Some scientists consider that PGCLCs and OLCs could be useful even if not 'perfect' because after transplantation in the seminiferous tubules or in the ovary they could recover the quality and capability to complete gametogenesis. Alternatively, they could be used as a source of parthenogenetic embryos for the production of ESC lines, as cytoplasmic donors in somatic cell nuclear transfer or to rejuvenate old oocytes.

Park and his co-workers demonstrated that ovarian cell-like cells differentiated from mouse SDSCs showed the robust ability to restore estradiol production and estrus cycling in ovariectomized mice resembling their normal counterparts.^45^ Although these results need to be reproduced in species with a gametogenesis more similar to humans, they represent promising news for women suffering from reduced estradiol production, a common phenomenon observed after menopause transition because of the exhaustion of the ovarian reserve.^84^ In this regard, transplantation of human SDSCs could be used for POF treatments.

Indeed, the most likely applications of PGCLCs and OLCs derived *in vitro* from various types of stem cells, including SDSCs, are in the area of research.^54, 85^ Lacking appropriate *in vitro* models for gametogenesis severely limits our knowledge regarding the molecular mechanisms governing such a fascinating and mysterious process. In particular, early gametogenesis has long been difficult to explore because of the inaccessibility of the embryo during early developmental stages (especially in human). A recent work by Irie *et al.*^86^ is a good example of the information that can be gained about the formation of the germline in humans using an *in vitro* system representing a period of embryo development inaccessible to experimentation. These authors showed that in the specification of PGCLCs, from human ESCs, SOX17 is the key regulator of the germline, whereas BLIMP1 represses endodermal and other somatic genes during specification of PGCLCs.

Paradoxically, it is just as well that the culture conditions fail to support efficient PGCLC differentiation into mature oocytes because this allows the identification of factors necessary to improve this process. Finally, many unanswered questions remain to be addressed regarding the biology and the characteristics of ASCs. Discovering how the culture system induces the differentiation of SDCSs into GCLCs could contribute to disclosing some of the stem cell secrets.

## Abbreviations

DS: dermal sheath
DP: dermal papilla
ORS: outer root sheath
IRS: inner root sheath
HS: hair shaft
NC: neural crest
SDSC: skin-derived stem cell
NCSC: neural crest stem cell
SKP: skin precursor cell
GCLC: germ cell-like cell
POF: premature ovarian failure
ASC: adult stem cell
ESC: embryonic stem cell
iPSC: induced pluripotent stem cell
PFF: porcine follicular fluid
PGC: primordial germ cell
PGCLC: primordial germ cell-like cell
EpiSC: epiblast stem cell
EpiLC: epiblast-like cell
EB: embryoid body
